# Individual, family, and social risk factors for child-to-parent violence in non-emancipated young adults

**DOI:** 10.3389/fpsyg.2026.1815327

**Published:** 2026-05-22

**Authors:** Jessica Burrai, Emanuela Mari, M. Carmen Cano-Lozano, María J. Navas-Martínez

**Affiliations:** 1 Department of Psychology, Sapienza University of Rome, Rome, Italy; 2 Department of Psychology, University of Jaén, Jaén, Spain

**Keywords:** child-to-parent violence, disciplinary strategies, family violence, impulsivity, young adults

## Abstract

**Objective:**

Child-to-parent violence (CPV) has been generally investigated during adolescence, whereas less attention has been paid to young adults who continue to live with their parents. Grounded in an ecological framework, this study aimed to examine how individual, familial, and social risk factors jointly relate to CPV toward mothers and fathers in a non-clinical sample of non-emancipated young adults, thereby extending existing evidence to a developmentally and culturally relevant population.

**Methods:**

Participants were 1,064 Italian young adults aged 18–25 years who had lived regularly with at least one parent during the previous year. They completed a self-report survey assessing CPV, anger, hostility, impulsivity, emotional dysregulation, substance use, direct family victimization, parental disciplinary strategies, perceived social support, and deviant peer affiliation. Spearman correlations and linear regression analyses were conducted separately for CPV toward mothers and fathers.

**Results:**

CPV toward mothers was positively associated with anger, hostility, impulsivity, emotional dysregulation (except for reduced self-awareness), substance use, direct family victimization, deviant peer affiliation, and disciplinary practices, and negatively associated with maternal discipline support and perceived social support. It was also positively associated with female gender. Similarly, CPV toward fathers showed positive associations with anger, hostility, impulsivity, emotional dysregulation, substance use, direct family victimization, deviant peer affiliation, and disciplinary practices, and negative associations with paternal discipline support and perceived social support, with no significant association with gender. Across models, family-related variables accounted for the largest proportion of explained variance.

**Conclusions:**

The findings indicate that CPV may persist into young adulthood and is strongly shaped by family dynamics and exposure to violence within the household. From a preventive and clinical perspective, interventions targeting emotional self-regulation and consistent parental disciplinary practices, while strengthening family support, may be particularly relevant during the transition to adulthood.

## 1 Introduction

The family environment is considered a safe space in which the bases for socialization are established and the first emotional bonds are formed. However, in some homes this dynamic is altered when children exercise violence toward their parents, inversing the usual direction of power relations within the family context. Child-to-parent violence (hereinafter CPV) refers to any act of physical, psychological, and/or financial violence by a child (son/daughter) toward a parent (Cottrell, 2001) that is carried out consciously, intentionally, and repeatedly over time (Pereira et al., 2017) seeks to obtain power, control (Cottrell, 2001), and domain (Howard and Rottem, 2008) over parental figures (Molla-Esparza and Aroca-Montolío, 2018) within the framework of habitual cohabitation (Ibabe, 2020). Despite being one of the least researched types of family violence, CPV—like any other form of violence—is now recognized as a serious public health problem (World Health Organization, 2002) that requires action from all sectors for its prevention.

Although most studies on CPV have focused on adolescence (Simmons et al., 2018), this phenomenon does not necessarily disappear over time but may persist or even intensify in later stages of development (Close et al., 2024; Ibabe, 2020). In Western societies characterized by late emancipation, parents and children continue to live together beyond adolescence (e.g., Eurostat, 2024), which extends the period during which family conflicts can develop and, in some cases, be expressed through violent behavior. Despite the relevance of the phenomenon, research on CPV in young adults (aged 18–25) is practically non-existent and constitutes an emerging research line in international literature (systematic review by Close et al., 2024).

The few studies using these samples have focused on analyzing the prevalence of CPV, warning of a high frequency of CPV in this age group, with percentages of occasional CPV of up to 85% in samples from Mexico, Spain, and Sweden (Cancino-Padilla et al., 2020; Cano-Lozano et al., 2021b; Johnson et al., 2022). In terms of repeated CPV, in Australia, up to 20% of young adults exert violence toward their father and 16% toward their mother (Simmons et al., 2020), while by type of CPV, in samples from Ecuador, Spain, and Italy (Burgos-Benavides et al., 2025; Ibabe et al., 2020; Navas-Martínez et al., 2025a) the percentages of physical CPV range between 2.5% and 5%, financial CPV range between 15% and 37%, psychological CPV from 60% to 67% and control and domain behaviors from 39% to 68%.

For all these reasons, the purpose of the present study was to examine the individual, familial, and social factors associated with CPV toward mothers and fathers among non-emancipated young adults living with their parents, within an ecological framework. Research has consistently shown that CPV is a multifactorial and multidimensional phenomenon (e.g., Cano-Lozano et al., 2023; Close et al., 2024; Del Hoyo-Bilbao et al., 2020; Simmons et al., 2018). The ecological model proposed by (Hong et al. 2012) provides a useful framework for organizing the risk factors involved in the development and maintenance of this type of violence in different systems (ontogenetic, microsystem, exosystem, and macrosystem). Along these lines, the nested ecological theory on CPV (Cottrell and Monk, 2004) highlights the role of the interaction between individual, family, and social risk factors, and suggests that, although the presence of a single factor belonging to any of these systems may be sufficiently relevant for the development of CPV, in general, the probability of CPV occurring is expected to increase as more risk factors accumulate in these ecological systems. The CPV studies have typically analyzed individual, familial, and social risk factors separately, focusing primarily on family-context variables, as this is the context in which such violence occurs, followed by individual variables and, to a lesser extent, social variables.

### 1.1 Research on individual risk factors

Individual risk factors refer to personal characteristics that may influence how children interpret and respond to social situations. Among the main individual risk factors for CPV are deficits in socio-cognitive processing, emotional difficulties, and substance use. Anger represents the affective component of aggression and refers to intense emotional arousal and a greater propensity to respond with anger to situations perceived as provocative or unfair (Buss and Perry, 1992). Studies with adolescents show that anger is positively related to CPV and is a consistent predictor of this type of violence (Calvete et al., 2015a; Orue et al., 2019), especially reactive CPV (Cano-Lozano et al., 2020, 2023; Contreras et al., 2020). (Simmons et al. 2020) found in a sample of young adults aged 18–24 that anger was a more characteristic variable of those who exercised CPV than of those who did not, and furthermore, it significantly predicted CPV.

Hostility is defined as a stable cognitive disposition characterized by negative attributions, resentment, and distrust toward others (Buss and Perry, 1992). For these authors, hostility represents the cognitive component of aggression and is another variable that has been linked to CPV in adolescents (Orue et al., 2019; Rosado et al., 2017) and has shown predictive capacity for this type of violence (Rosado et al., 2017) among the ages of 13 and 18, particularly reactive CPV (Cano-Lozano et al., 2020). However, hostility did not predict CPV in the group of young adults aged 19–21 in the study by (Rosado et al. 2017), so the role of this variable in samples of young adults is still unclear.

Another key variable of CPV is impulsivity, understood as a personality trait that reflects difficulties in inhibiting responses and maintaining control over thoughts and behavior (Barratt and Patton, 1983). High levels of impulsivity have been linked to increased CPV in adolescents (Calvete et al., 2011; Del Hoyo-Bilbao et al., 2020), standing out as one of the most relevant individual predictors of CPV (Del Hoyo-Bilbao et al., 2020; Rico et al., 2017). The above results have evaluated the motor dimension of impulsivity, referring to the tendency to act precipitously or to a reduced capacity for behavioral self-control in response to internal or external stimuli (Patton et al., 1995). Other dimensions of impulsivity, such as the cognitive dimension, have been less researched in the field of CPV, although they have also shown predictive power (Rico et al., 2017). This type of impulsivity refers to instability and speed in mental processing and the tendency to make unplanned decisions (Patton et al., 1995). However, the specific role of cognitive impulsivity in young adults is unknown.

Regarding emotional variables, studies have found that adolescents who engage in CPV often have difficulty identifying, expressing, and controlling their emotions (Beckmann et al., 2021; Martínez-Ferrer et al., 2018), as well as negative relationships between emotional intelligence and CPV (López-Martínez et al., 2019; Navas-Martínez and Cano-Lozano, 2023). These studies also showed that emotional intelligence negatively predicts CPV, especially low levels of emotional regulation (Navas-Martínez and Cano-Lozano, 2023). Difficulties in emotional regulation comprise up to five different processes (Gratz and Roemer, 2004): lack of emotional acceptance (difficulty accepting one's own discomfort), interference of negative emotions in goal achievement (difficulty orienting behavior toward goals in the face of negative emotions), lack of control in emotional regulation or emotional impulsivity (difficulty to regulate negative emotions and to inhibit impulses in intense emotional situations), lack of emotional clarity (difficulty identifying one's own emotions) and lack of emotional awareness (difficulty attending to internal emotional states). In samples of young adults, only emotional impulsivity has been analyzed (Simmons et al., 2020) found that those who exercised CPV toward their mother had higher levels of emotional impulsivity than those who did not exercise CPV. However, they found no differences between young adults who exercised and did not exercise CPV toward their father, nor did they find any predictive capacity of this variable on CPV.

Finally, drug use has also been consistently linked to CPV in adolescent samples (Beckmann et al., 2021; Calvete et al., 2011; Cano-Lozano et al., 2020; Del Hoyo-Bilbao et al., 2020; Ibabe et al., 2013; Noh-Moo et al., 2023) and has been identified as another of the most relevant individual predictors of CPV (Del Hoyo-Bilbao et al., 2020; Noh-Moo et al., 2023; Rosado et al., 2017), both reactive and instrumental (Cano-Lozano et al., 2020). In young adults' samples, the results remain inconsistent. This variable did not significantly predict CPV in the 19–21 age group (Rosado et al., 2017), although the use of new psychoactive substances did significantly predict CPV in the study by (Johnson et al. 2022).

### 1.2 Research on family risk factors

Among the main family risk factors, exposure to violence at home stands out, referring to situations in which children suffer violence from their parents (direct family victimization) and/or are exposed to violence between their parents (vicarious family victimization). In adolescents, both types of victimization have been consistently found to be related to and predictive of CPV (meta-analysis by Gallego et al., 2019). However, is the direct type of family victimization that stand out as one of the main predictors of this type of violence in numerous studies (e.g., Beckmann, 2020; Cano-Lozano et al., 2024; Del Hoyo-Bilbao et al., 2020; Navas-Martínez et al., 2025b; Izaguirre and Calvete, 2017). Studies on CPV that have delved into this type of family victimization have found a certain correspondence between the gender of the victimizing parent and the gender of the parent toward whom the CPV is directed. Specifically, CPV toward the father is mainly explained by the victimization of the father, while CPV toward the mother is mainly explained by the victimization of the mother, but also by that of the father (Cano-Lozano et al., 2024; Navas-Martínez and Cano-Lozano, 2022). In samples of young adults, although smaller, the evidence shows results that point in the same direction. Specifically, young adults who exercise CPV are more exposed to direct victimization at home (Simmons et al., 2020). In addition, this type of victimization is significantly related to and predict CPV (Cano-Lozano et al., 2021b; Simmons et al., 2020).

Some variables related to parenting styles also play a significant role in the development of CPV in adolescent samples. For example, parental ineffectiveness, referring to practices that do not produce the desired result (Baumrind, 1997), has been positively related to CPV (Del Hoyo-Bilbao et al., 2020) and to difficulty in controlling children's misbehavior (Calvete et al., 2015b). Parental stress, referring to the overload that parents experience due to the responsibilities of parenting (Abidin, 1992), has also been positively related to CPV (Aroca-Montolío et al., 2014; Brezina, 1999; Del Hoyo-Bilbao et al., 2020), as well as parental impulsivity in exercising discipline (Del Hoyo-Bilbao et al., 2020). Parental support and affection, referring to the provision of warmth and sensitivity to the child's needs, has been negatively related to CPV (Rohner, 1986), being another of the most relevant family predictors of this type of violence in numerous studies (Bautista-Aranda et al., 2024; Cano-Lozano et al., 2020; Ibabe et al., 2013; Zhang et al., 2019). In samples of young adults, only (Camargo 2023) has analyzed variables related to discipline, and only in relation to impulsivity and affection of the mother and CPV toward the mother. She found that CPV toward the mother was positively related to maternal impulsivity and negatively related to maternal affection, while only impulsivity significantly predicted CPV.

### 1.3 Research on social risk factors

Regarding social variables, various studies involving samples of adolescents show an association between CPV and deviant peer groups (Baker and Bonnick, 2021; Calvete et al., 2011; Cano-Lozano et al., 2020), referring to a group of people of similar age who engage in problematic behaviors, substance use, vandalism, violence, etc. However, the effect of these groups on CPV may not be direct but rather mediated by other variables such as social support. Specifically, (Del Hoyo-Bilbao et al. 2020) found that parental support was negatively related to the deviant peer group, and that the influence of the deviant peer group on CPV occurred mainly through drug use.

Perceived social support, comprising three dimensions (support from family, friends, and significant others; Zimet et al., 1988), is defined as the emotional support and resources that a person receives from their environment (Williams and Galliher, 2006). In the context of CPV, this variable has been analyzed mainly in samples of parents and in general terms, without distinguishing between its specific dimensions. For example, (Herrador et al. 2017) observed that mothers who experienced CPV perceived lower levels of social support than those who did not experience CPV, while in (Bautista-Aranda et al. 2025), also in a sample of parents, perceived social support was negatively related to CPV and predicted this type of violence. Studies on other types of violence show negative relationships between perceived social support and, for example, the perpetration of cyberbullying (Heiman and Olenik-Shemesh, 2017; Yang et al., 2021), as well as the protective role of this variable in juvenile violence in general (Walsh, 2023).

### 1.4 The present study

The review shows that the evidence on risk factors for CPV comes mainly from adolescent samples, with research on young adults still limited, especially about the study of risk factors for CPV at this stage of development. Several studies suggest that the risk factors associated with CPV may vary as children transition into emerging adulthood, due to changes in their level of maturity and family living patterns (Ibabe, 2020; Simmons et al., 2018). However, to date, studies on CPV with samples of young adults have focused on analyzing the prevalence of CPV (Burgos-Benavides et al., 2025; Camargo, 2023; Cano-Lozano et al., 2021b; Ibabe et al., 2020; Johnson et al., 2022; Navas-Martínez et al., 2025a; Sheed et al., 2024; Simmons et al., 2020), and only a few have incorporated the analysis of certain individual and family variables (Camargo, 2023; Cano-Lozano et al., 2021b; Ibabe et al., 2020; Simmons et al., 2020).

The scarcity and fragmentation of available evidence still do not allow us to establish the psychosocial profile of young adults involved in CPV, which is essential for the implementation of effective interventions in this age group. Therefore, the present study aims to analyze the individual, family, and social profile associated with CPV in a population of non-emancipated young adults, contributing to filling a significant gap in the literature. Furthermore, this contribution is particularly relevant in the Italian context, a country that, in addition to being among the top five countries with the highest rates of late emancipation in Europe (Eurostat, 2024), has hardly any previous research in this area despite the high prevalence recorded (Navas-Martínez et al., 2025a,c; Sicurella, 2020). The following research questions were formulated:

RQ1. Analyse the relationship between CPV toward the father and toward the mother and a series of individual variables (anger, hostility, motor impulsivity, cognitive impulsivity, difficulties in emotional regulation, and drug use), family variables (direct victimization at home and parental discipline), and social variables (perceived social support and deviant peer group). Significant relationships are expected between CPV and individual variables (Rico et al., 2017; Rosado et al., 2017; Simmons et al., 2020), family variables (Camargo, 2023; Cano-Lozano et al., 2021b; Ibabe et al., 2020; Simmons et al., 2020), and social variables (Bautista-Aranda et al., 2025; Del Hoyo-Bilbao et al., 2020).

RQ2. To examine the role of individual, family, and social variables in predicting CPV toward fathers and mothers. Anger (Simmons et al., 2020), motor and cognitive impulsivity (Rico et al., 2017), drug use (Johnson et al., 2022), direct victimization at home (Camargo, 2023; Cano-Lozano et al., 2021b; Simmons et al., 2020), and parental impulsivity (Camargo, 2023) are expected to be significant predictors of CPV. No predictions are made for the other variables, given the lack of evidence in the youth population.

## 2 Methods

### 2.1 Procedure

A convenience sample was invited to participate in our anonymous online survey conducted using the Qualtrics platform. The questionnaire was distributed via social media platforms (i.e., Facebook, Instagram), instant messaging services, and official university channels. Eligibility criteria required participants to be between 18 and 25 years old, to have Italian nationality or reside in Italy, and that they had lived regularly with their father and/or mother during the last year. Anonymity was ensured, and participants could refuse to give consent or withdraw from the study at any time. All study procedures were conducted in accordance with the Declaration of Helsinki and were approved by the Institutional Review Board of the Department of Psychology at the University of Rome “Sapienza” (protocol number 0001707, [UOR: SI000030-II/23]) and from the Ethics Committee of the University of Jaén [protocol number: OCT.19/1.PRY reference: ABR.22/5.PRY].

### 2.2 Participants

The final sample consisted of 1,064 participants, of whom 41.5% were male (*n* = 442), with ages ranging from 18 to 25 years (*M*_*age*_ = 20.63; *SD* = 2.00). Two participants selected non-binary/other; given the very small size of this subgroup, they were not included in analyses involving gender. Therefore, gender was operationalized as a binary variable for analytical purposes. All socio-demographic characteristics of the participants are reported in Table 1. As shown in Table 1, the sample was predominantly female, and most participants reported that their parents were married. A smaller proportion indicated that their parents were separated or divorced, while cohabitation, widowhood, and parents who had never lived together were less frequently reported. Regarding socioeconomic status, the majority of participants reported a monthly family income between €1,000 and €3,000, with a relatively balanced distribution across income categories above €2,000. In terms of educational level, most participants had completed secondary or higher education, with a substantial proportion reporting university-level qualifications, including bachelor's, master's, and postgraduate degrees.

**Table 1:** Socio-demographic characteristics of the sample.

Demographic variables	*n* (%)
Gender	
Male	442 (41.50)
15.6-7.4,-1.3242ptFemale	622 (58.50)
Marital status of their parents	
Married	770 (72.40)
Cohabitation	35 (3.30)
Separated/Divorced	223 (21.00)
Widowed	30 (2.80)
15.6-7.4,-1.3242pt Never lived together	6 (0.60)
Monthly income	
< 1,000€	45 (4.20)
1,000€−2,000€	323 (30.40)
2,000€−3,000€	355 (33.40)
15.6-7.4,-1.3242pt >3,000€	341 (32.00)
Education	
Nothing	4 (0.40)
Primary studies	13 (1.20)
Secondary studies	143 (13.40)
Higher studies	548 (51.50)
Bachelor's degree	89 (8.40)
Master's degree	174 (16.40)
Postgraduate studies	93 (8.70)

*N* = 1,064.

Participants were included in the study if they reported having lived with at least one parent in the past year, based on the question: “In the past year, have you lived with your mother and/or your father?” Therefore, the total sample consists exclusively of individuals who met this criterion.

Participants were then asked to indicate their current living arrangement. Specifically, 182 participants reported living with only their mother, 32 with only their father, 37 with their mother and her partner, and 9 with their father and his partner, while the majority of the sample (*n* = 792) reported living with both parents. The 12 remaining participants had changed their living arrangements at the time of completing the questionnaire and were no longer living with their parents, having moved to live with other individuals, either family members or non-family members. However, during the previous year, they had lived with at least one parent.

### 2.3 Instruments

Brief sociodemographic *ad hoc* questionnaire: includes a series of questions aimed at gathering sociodemographic information about the participants, including gender (male, female, non-binary, other), age (in years), nationality, current educational status (e.g., “If you are currently studying, please indicate the type of studies you are attending”), monthly household income (assessed through approximate ordinal income categories), and marital status (e.g., single, in a relationship, married, separated/divorced).

The Child-to-Parent Violence Questionnaire, youth version (CPV-Q, Cano-Lozano et al., 2021a; Italian validation by Navas-Martínez et al., 2025a). The CPV-Q consists of 19 items investigating the frequency of four types of violence toward parents (For each dimension, the following statements represent example items of the scale: Psychological: “I have threatened my parents (with hurting them, with hurting myself, with running away from home)”; Physical: “I have kicked, slapped, and/or punched my parents”; Financial: “I have stolen money from my parents”; Control/domain: “I have imposed on my parents that at home they do what I want”) in the last year. The scale assesses violence toward fathers (α = 0.79) and mothers (α = 0.82) separately, using a five-point Likert scale ranging from “never” to “very often = six times or more.”

#### 2.3.1 Individual variables

Aggression Questionnaire (AQ, Buss and Perry, 1992; Italian validation by Fossati et al., 2003). Includes 29 items assessing four dimensions of aggression: Physical Aggression (nine items), Verbal Aggression (five items), Anger (seven items), and Hostility (eight items). The total score ranges from 29 to 145. Subscale scores are calculated by summing the respective item scores. In this study, the Anger (α = 0.75) and Hostility (α = 0.78) subscales were used. Responses were given on a five-point scale ranging from “completely false” to “completely true” (e.g., “I get angry quickly, but I calm down just as fast”).

Barratt Impulsiveness Scale (BIS-11, Patton et al., 1995; Italian validation by Fossati et al., 2001). The BIS-11 is a 30-item self-report questionnaire designed to assess impulsiveness. Items are rated on a four-point scale from “rarely/never” to “almost always/always.” A higher total score indicates higher levels of impulsiveness. Some items are reverse scored to reduce response bias. In this study, 20 items were selected from the original scale, corresponding to the Motor Impulsivity (α = 0.65) and Cognitive Impulsivity (α = 0.73) subscales (e.g., “I do things without thinking”).

Difficulties in Emotion Regulation Scale, Short Form (DERS-20, Gratz and Roemer, 2004; Italian validation by Lausi et al., 2020). It assesses five dimensions of emotion regulation: Non-acceptance, Goals, Impulse, Clarity, and Awareness. In this study, the Italian 20-item short form developed by Lausi et al. was used. Items are rated on a five-point scale from “almost never” to “almost always,” with higher scores indicating greater difficulty in emotion regulation. The dimensions and Cronbach's alpha of the scale are as follows: Lack of acceptance = 0.89; Difficulties in distraction (Goals) = 0.89; Lack of control (Impulse) = 0.91; Difficulties in recognition (Clarity) = 0.83; Reduced self-awareness = 0.83 (e.g., “I pay attention to how I feel”).

Tobacco, Alcohol, and Other Drug Use Scale (ESCON, García del Castillo-López, 2011, p. 10). Consisting of nine items, was created to evaluate how often participants used various substances over the last year. The items were rated on a five-point Likert scale ranging from “never” to “daily”; Cronbach's alpha was 0.56. The low internal consistency of the drug use scale can be attributed to the limited number of items, which is typical of *ad hoc* measures designed for specific and preliminary assessments (e.g., “Please indicate how often you used tobacco during the past year”).

#### 2.3.2 Family variables

Exposure to Violence Scale, Home subscale (EEV; Orue and Calvete, 2010). The EEV is an instrument designed to assess adolescents' exposure to violence in different contexts. In this study, only the “home” subscale was used. An adaptation of the scale was conducted to evaluate the frequency of the violence experienced from the father and the mother in the last year through six items (maternal violence α = 0.71, paternal violence α = 0.69) with a five-point Likert scale ranging from “never” to “very often,” with higher scores indicating greater direct victimization at home (e.g., “How often did your father yell at you or insult you?”).

Dimensions of Discipline Inventory, Section D (DDI-D; Straus and Fauchier, 2007). It evaluates the perception of how parental discipline was implemented during adolescence (12–17 years old) by the father and the mother using a five-point scale ranging from “never” to “always or almost always,” through 25 items. In this study, the following subscales from Section D were used: Parental distress: α = 0.60 (mother), α = 0.61 (father); Parental impulsiveness: α = 0.85 (mother), α = 0.90 (father); Support and affection: α = 0.81 (mother), α = 0.86 (father) (e.g., “Your parents got very angry when you misbehaved”).

#### 2.3.3 Social variables

Multidimensional Scale of Perceived Social Support (MSPSS; Zimet et al., 1988, Italian validation by Di Fabio and Palazzeschi, 2015). The MSPSS is a 12-item self-report questionnaire designed to assess perceived social support from three sources in the last year: family (α = 0.83), friends (α = 0.88), and significant others (α = 0.87). Each item is rated on a seven-point Likert scale ranging from “very strongly disagree” to “very strongly agree,” with higher scores indicating greater perceived support (e.g., “There is a special person with whom I can share my sorrows and my joys”).

Deviant Peer Group, *Ad hoc* scale (based on Peers' Deviation Scale by Barnow et al., 2005). An *ad hoc* three-item scale was developed to assess the presence of deviant peers in the participant's peer group in the last year, based on the framework proposed by (Barnow et al. 2005). The sum of the three items provides a total score indicating the extent of deviant behavior among the participants' peers; Cronbach's alpha was 0.59. The low internal reliability of the deviant peer's scale is likely due to the brevity of the instrument, which includes only a few items, a common characteristic of *ad hoc* scales used in exploratory studies (e.g., “Think about your group of friends over the past year and indicate how many of them were involved in criminal activities”).

All instruments used in the present study have shown adequate evidence of construct validity in previous research, including factorial validity and convergent validity, as reported in the respective validation studies.

### 2.4 Data analysis

Statistical analyses were conducted using IBM SPSS Statistics (version 27). As a first step, descriptive statistics (means and standard deviations) were computed for each scale. The assumption of normality was assessed by examining skewness and kurtosis indices.

To examine the presence of significant relationships between child-to-parent violence (CPV)—toward the mother and toward the father—and the individual and family variables considered in the study, bivariate correlations were performed using Spearman's rho coefficient, as several variables showed non-normal distributions.

Finally, to identify the predictors of CPV, two separate standard multiple linear regression analyses (enter method) were conducted: one for CPV toward the mother and one for CPV toward the father. All predictors were entered simultaneously in each model. Results were considered statistically significant at *p* < 0.05.

Additionally, an a priori power analysis was conducted using G^*^Power (version 3.1) to determine the required sample size for a multiple linear regression model. Assuming a small-to-medium effect size (Cohen's *f*^2^ = 0.10), an alpha level of 0.05, a desired statistical power (1 – β) of 0.80, and 24 predictors, the analysis indicated a minimum required sample size of 219 participants. Given that the final sample size (*n* = 1,064) substantially exceeded this minimum requirement, the study was adequately powered to detect even relatively small proportions of explained variance in the dependent variable. This supports the claim that the sample size was sufficient to identify statistically significant effects within the regression model.

## 3 Results

The descriptive statistics of the study variables are reported in Table 2.

**Table 2:** Descriptive statistics.

Variable	Mean	*SD*	Skewness	*SE*	Kurtosis	*SE*
Child-to-Mother violence	9.08	7.14	2.00	0.08	5.97	0.15
Child-to-Father violence	7.68	6.44	2.06	0.08	7.21	0.15
Individual variables						
AQ_Anger	19.37	4.95	0.10	0.08	−0.54	0.15
AQ_Hostility	22.34	5.86	−0.02	0.08	−0.37	0.15
BIS_Motor impulsivity	19.12	3.90	0.61	0.08	0.23	0.15
BIS_Cognitive impulsivity	18.37	3.97	0.57	0.08	0.20	0.15
DERS_Lack of acceptance	10.74	5.14	0.88	0.08	−0.02	0.15
DERS_Difficulties in distraction	12.09	4.17	0.21	0.08	−0.96	0.15
DERS_Lack of control	7.90	3.94	1.12	0.08	0.48	0.15
DERS_Difficulties in recognition	7.22	2.96	0.66	0.08	−0.29	0.15
DERS_Reduced self-awareness	14.89	3.49	−0.62	0.08	−0.11	0.15
15.6-7.4,-1.3498pt ESCON	5.34	3.72	1.13	0.08	3.56	0.15
Family variables						
EEV_Father	1.78	1.91	1.48	0.08	2.45	0.15
EEV_Mother	2.10	2.06	1.40	0.08	2.18	0.15
DDI_Inefficiency_Father	2.78	2.44	1.02	0.08	0.71	0.15
DDI_Inefficiency_Mother	2.88	2.49	1.07	0.08	0.87	0.15
DDI_DiStress_Father	2.80	1.91	0.47	0.08	−0.36	0.15
DDI_DiStress_Mother	3.36	1.96	0.31	0.08	−0.56	0.15
DDI_Impulsivity_Father	1.52	2.06	1.42	0.08	1.26	0.15
DDI_Impulsivity_Mother	1.59	1.93	1.30	0.08	1.17	0.15
DDI_Support_Father	7.79	3.34	−0.70	0.08	−0.39	0.15
DDI_Support_Mother	8.49	2.88	−0.90	0.08	0.33	0.15
Social variables						
Deviant Peer Group	0.88	1.14	1.68	0.08	3.33	0.15
MSPSS_Family	18.82	4.46	−0.94	0.08	0.64	0.15
MSPSS_Friends	19.15	4.51	−1.03	0.08	1.05	0.15
MSPSS_Significant others	19.82	4.66	−1.17	0.08	1.01	0.15

DERS, Difficulties in emotion regulation; ESCON, substance use; EEV, direct family victimization; DDI, parental discipline; MSPSS, perceived social support.

Results are presented according to the two research questions. First, associations among variables (RQ1) are reported; second, the results of the predictive models (RQ2) are described.

### 3.1 Correlation analyses

To examine the presence of significant relationships between CPV toward the father and the mother and the individual, family, and social variables considered in the study, bivariate correlations were conducted using Spearman's rho coefficient. Effect sizes were interpreted according to empirically derived benchmarks (Bosco et al., 2015), whereby correlations are classified as small (|*r*| < 0.09), medium (0.09 ≤ |*r*| ≤ 0.26), and large (|*r*| > 0.26).

Regarding individual variables, CPV toward both parents was positively associated with anger, hostility, impulsivity, emotional dysregulation, and substance use, with effect sizes generally in the medium range, and reaching the large range for anger (mother: *r* = 0.326) and lack of emotional control (mother: *r* = 0.295). No significant associations emerged for reduced self-awareness. Gender was weakly associated only with CPV toward the mother (medium effect).

With respect to family variables, CPV showed the strongest associations overall. Direct family victimization and dysfunctional disciplinary practices (inefficiency, distress, and impulsivity) were consistently and positively related to CPV, with medium-to-large effect sizes. Several associations fell within the large range, including victimization (mother: *r* = 0.518; father: *r* = 0.497), disciplinary inefficiency (mother: *r* = 0.433; father: *r* = 0.382), parental distress (mother: *r* = 0.417; father: *r* = 0.303), and parental impulsivity (mother: *r* = 0.435; father: *r* = 0.354). Conversely, parental support was negatively associated with CPV, with medium effect sizes.

Finally, regarding social variables, CPV toward both parents was positively associated with deviant peer group (medium effects) and negatively associated with perceived social support from family, friends, and significant others, with effect sizes ranging from small to medium and reaching the large range for family support in relation to CPV toward the mother (*r* = −0.276). The correlation analyses are reported in Table 3.

**Table 3:** Correlation between child-to-parent violence and individual, family and social variables.

Individual variables	Child-to-mother violence	Child-to-father violence
AQ_Anger	0.326^***^	0.213^***^
AQ_Hostility	0.253^***^	0.171^***^
BIS_Motor impulsivity	0.254^***^	0.235^***^
BIS_Cognitive impulsivity	0.177^***^	0.162^***^
DERS_Lack of acceptance	0.140^***^	0.123^***^
DERS_Difficulties in distraction	0.191^***^	0.177^***^
DERS_Lack of control	0.295^***^	0.219^***^
DERS_Difficulties in recognition	0.136^***^	0.090^**^
DERS_Reduced self-awareness	0.013	0.023
ESCON	0.245^***^	0.181^***^
15.6-7.4,-1.3498pt Gender	0.108^***^	0.038
Family variables		
EEV_Father	0.351^***^	0.497^***^
EEV_Mother	0.518^***^	0.305^***^
DDI_Inefficiency_Father	0.320^***^	0.382^***^
DDI_Inefficiency_Mother	0.433^***^	0.302^***^
DDI_DiStress_Father	0.226^***^	0.303^***^
DDI_DiStress_Mother	0.417^***^	0.283^***^
DDI_Impulsivity_Father	0.253^***^	0.354^***^
DDI_Impulsivity_Mother	0.435^***^	0.335^***^
DDI_Support_Father	−0.186^***^	−0.167^***^
15.6-7.4,-1.3498pt DDI_Support_Mother	−0.199^***^	−0.136^***^
Social variables		
Deviant Peer Group	0.184^***^	0.124^***^
MSPSS_Family	−0.276^***^	−0.223^***^
MSPSS_Friends	−0.123^***^	−0.068^*^
MSPSS_Significant others	−0.139^***^	−0.103^**^

DERS, Difficulties in emotion regulation; ESCON, substance use; EEV, direct family victimization; DDI, parental discipline; MSPSS, perceived social support.

^*^p < 0.05,

^**^p < 0.01, and

^***^p < 0.001.

### 3.2 Regression analyses

Standard linear regressions were performed separately for fathers and mothers to identify predictors of CPV across individual, family, and social variables. The proposed model included all predictors, and tolerance and VIF indices indicated no multicollinearity issues. The analytic sample was consistent across regression models (*N* = 1,064). The number of predictors differed slightly between models, as gender was included only in the model predicting CPV toward the mother, resulting in 24 predictors for the maternal model and 23 for the paternal model.

For CPV toward the mother (see Table 4), the model explained 44.8% of the variance (*F*_(24, 1, 039)_ = 35.121, *p* < 0.001). The strongest predictors were family-related variables, particularly direct victimization by the mother (β = 0.274, *p* < 0.001) and ineffective maternal disciplinary strategies (β = 0.198, *p* < 0.001). CPV toward the mother was also positively predicted by supportive (β = 0.133, *p* < 0.001) and impulsive maternal disciplinary strategies (β = 0.120, *p* = 0.002), substance use (β = 0.121, *p* < 0.001), female gender (β = 0.088, *p* < 0.001), motor impulsivity (β = 0.082, *p* = 0.003), direct victimization by the father (β = 0.080, *p* = 0.016), and peer deviance (β = 0.062, *p* = 0.024). In contrast, perceived family social support was negatively associated with CPV (β = −0.150, *p* < 0.001).

**Table 4:** Regressions models for child-to-mother violence.

Predictors	Coefficient					Model summary				
	*B*	*SE*	β	*t*	*p*	*R* ^2^	*R*^2^Adj	*F*	*df*1; *df*2	*p*
Individual variables										
AQ_Anger	0.810	0.046	0.056	1.759	0.079	0.448	0.435	35.121	24; 1,039	< 0.001
AQ_Hostility	0.040	0.037	0.033	1.102	0.271					
BIS_Motor impulsivity	0.150	0.050	0.083	3.010	**0.003**					
BIS_Cognitive impulsivity	−0.032	0.053	−0.018	−0.597	0.550					
DERS_Lack of acceptance	0.005	0.040	0.004	0.132	0.895					
DERS_Difficulties in distraction	0.042	0.052	0.024	0.802	0.422					
DERS_Lack of control	0.067	0.062	0.037	1.086	0.278					
DERS_Difficulties in recognition	−0.114	0.067	−0.047	−1.687	0.092					
ESCON	0.233	0.052	0.121	4.494	**< 0.001**					
Gender	1.271	0.364	0.088	3.497	**< 0.001**					
Family variables										
EEV_Father	0.299	0.123	0.080	2.419	**0.016**					
EEV_Mother	0.950	0.115	0.274	8.272	**< 0.001**					
DDI_Inefficiency_Father	−0.106	0.123	−0.036	−0.865	0.387					
DDI_Inefficiency_Mother	0.568	0.126	0.198	4.499	**< 0.001**					
DDI_DiStress_Father	−0.053	0.144	−0.014	−0.366	0.715					
DDI_DiStress_Mother	−0.012	0.144	−0.003	−0.086	0.931					
DDI_Impulsivity_Father	0.095	0.139	0.027	0.683	0.495					
DDI_Impulsivity_Mother	0.445	0.143	0.120	3.116	**0.002**					
DDI_Support_Father	−0.045	0.069	−0.021	−0.647	0.518					
DDI_Support_Mother	0.329	0.080	0.133	4.091	**< 0.001**					
Social variables										
Deviant Peer Group	0.390	0.173	0.062	2.261	**0.024**					
MSPSS_Family	−0.241	0.048	−0.150	−4.979	**< 0.001**					
MSPSS_Friends	0.017	0.044	0.011	0.396	0.692					
MSPSS_SignificantOthers	−0.022	0.043	−0.014	−0.515	0.606					

*N* = 1,064. Gender 1, female; DERS, Difficulties in emotion regulation; ESCON, substance use; EEV, direct family victimization; DDI, parental discipline; MSPSS, perceived social support. Bold values indicate statistically significant results at *p* < 0.05.

Regarding CPV toward the father (see Table 5), the model explained 36.6% of the variance [*F*_(23, 1, 040)_ = 26.118, *p* < 0.001]. Again, family-related variables emerged as the strongest predictors, especially direct victimization by the father (β = 0.386, *p* < 0.001) and ineffective paternal disciplinary strategies (β = 0.301, *p* < 0.001). Additional positive predictors included maternal impulsive disciplinary strategies (β = 0.198, *p* < 0.001), substance use (β = 0.111, *p* < 0.001), and motor impulsivity (β = 0.085, *p* = 0.003). Conversely, maternal ineffective disciplinary strategies (β = −0.169, *p* < 0.001), perceived family social support (β = −0.079, *p* = 0.015), and difficulties in emotion recognition (β = −0.063, *p* = 0.035) were negatively associated with CPV toward the father.

**Table 5:** Regressions models for child-to-father violence.

Predictors	Coefficient					Model summary				
		*B*	*SE*	β	*t*	*p*	*R* ^2^	*R*^2^Adj	*F*	*df*1; *df*2	*p*
Individual variables										
AQ_Anger	0.061	0.044	0.047	1.383	0.167	0.366	0.352	26.118	23; 1,040	< 0.001
AQ_Hostility	0.041	0.035	0.037	1.156	0.248					
BIS_Motor impulsivity	0.141	0.048	0.085	2.935	**0.003**					
BIS_Cognitive impulsivity	0.040	0.051	0.025	0.779	0.436					
DERS_Lack of acceptance	0.006	0.039	0.005	0.148	0.883					
DERS_Difficulties in distraction	0.017	0.050	0.011	0.331	0.740					
DERS_Lack of control	0.043	0.060	0.026	0.724	0.469					
DERS_Difficulties in recognition	−0.137	0.065	−0.063	−2.107	**0.035**					
ESCON	0.192	0.050	0.111	3.849	**< 0.001**					
Family variables										
EEV_Father	1.305	0.119	0.386	10.932	**< 0.001**					
EEV_Mother	−0.122	0.111	−0.039	−1.098	0.273					
DDI_Inefficiency_Father	0.794	0.119	0.301	6.696	**< 0.001**					
DDI_Inefficiency_Mother	−0.437	0.122	−0.169	−3.585	**< 0.001**					
DDI_DiStress_Father	−0.211	0.139	−0.063	−1.522	0.128					
DDI_DiStress_Mother	0.011	0.140	0.003	0.079	0.937					
DDI_Impulsivity_Father	−0.069	0.134	−0.022	−0.517	0.605					
DDI_Impulsivity_Mother	0.661	0.138	0.198	4.789	**< 0.001**					
DDI_Support_Father	0.123	0.067	0.064	1.830	0.067					
DDI_Support_Mother	0.081	0.078	0.036	1.047	0.295					
Social variables										
Deviant Peer Group	0.080	0.163	0.014	0.489	0.625					
MSPSS_Family	−0.114	0.047	−0.079	−2.438	**0.015**					
MSPSS_Friends	0.031	0.042	0.022	0.725	0.468					
MSPSS_Significant Others	−0.030	0.041	−0.022	−0.738	0.461					

*N* = 1,064. DERS, Difficulties in emotion regulation; ESCON, substance use; EEV, direct family victimization; DDI, parental discipline; MSPSS, perceived social support. Bold values indicate statistically significant results at *p* < 0.05.

## 4 Discussion

The present study aimed to examine how individual, familial, and social risk factors are associated in shaping episodes of child-to-parent violence (CPV), focusing on a group of non-emancipated Italian young adults still living with at least one parent. This group represents a segment of the population that is rarely investigated in CPV research, even though prolonged cohabitation with parents is a common feature of Italian society (Eurostat, 2024). By analyzing multiple ecological levels together, this study sought to capture a more nuanced picture of the circumstances that may sustain or trigger CPV during the transition to adulthood.

Overall, this research addresses two key gaps in the literature: the lack of studies on CPV among non-emancipated young adults and the absence of evidence from Mediterranean contexts.

By integrating individual, familial, and social dimensions within a single framework, the study contributes to a more context-sensitive and developmentally grounded understanding of CPV and offers new insights for prevention and intervention strategies that take cultural realities into account.

With respect to the first research question (RQ1), the findings confirm the existence of significant associations between CPV and a range of risk factors across the three ecological levels considered: individual, familial, and social (Cottrell and Monk, 2004).

(1) At the individual level, the data indicate that motor impulsivity, hostility, anger, and substance use are associated with a higher likelihood of violent acts toward both parents, in line with previous research (Rico et al., 2017; Rosado et al., 2017; Simmons et al., 2020). These findings suggest that difficulties in emotional and behavioral self-regulation may represent a critical vulnerability in CPV, confirming the role of impulse control deficits, hostility, anger, and exposure to high-risk contexts in promoting violent behaviors. Hostility and anger may contribute to the association between impulsivity and violent behavior, reflecting difficulties in modulating emotional responses and delaying gratification. From this perspective, aggressive behaviors can be interpreted as a dysfunctional strategy for managing frustration and family conflicts, reinforcing the hypothesis of a temperamental vulnerability that, in the absence of adequate regulatory resources, may escalate into episodes of violence toward parents.

(2) At the familial level, direct victimization—being victims of parental violence—and the perception of parental inefficacy and impulsivity, both maternal and paternal, were strongly correlated with CPV. Conversely, perceived support from both parents was negatively associated with violent behaviors, suggesting a potential protective role. Overall, these findings provide support for the hypothesis of an intergenerational cycle of violence (Camargo, 2023; Cano-Lozano et al., 2021b; Ibabe et al., 2020; Simmons et al., 2020), which appears to persist into young adulthood, where experiences of direct victimization and inconsistent parental practices may contribute to maintaining dysfunctional and conflictual relational dynamics.

(3) At the social level, a significant association emerged between affiliation with deviant peers and CPV toward both parents, while perceived social support—derived not only from the family and friends but also from other significant figures—was confirmed as an important protective factor. However, the influence of peer groups appeared less pronounced than that reported in studies with adolescent samples (Calvete et al., 2011; Del Hoyo-Bilbao et al., 2020). This difference may be attributable to the specificities of the Italian context, where prolonged cohabitation with the family of origin tends to maintain a central role for familial relationships, reducing the weight of peers in shaping behavioral models and conflict dynamics.

Overall, the results related to the first objective also confirm the multidimensional nature of the phenomenon: CPV cannot be understood through a single explanatory dimension but emerges from the complex and reciprocal interaction among individual characteristics, family dynamics, and social influences, which together may contribute to shaping violent behavior in young adulthood. According to empirically derived benchmarks (Bosco et al., 2015), the observed correlations generally fall within the small-to-medium range, with several associations reaching large effect sizes, particularly for family-related variables such as direct victimization, which showed the strongest relationships.

Regarding the second research question (RQ2), the predictive models allowed the identification of key variables that significantly contribute to explaining CPV.

(1) At the individual level, motor impulsivity and substance use were confirmed as significant predictors of CPV toward both parents, suggesting that difficulties in impulse control and exposure to high-risk contexts are central components of violent behavior (Rico et al., 2017; Johnson et al., 2022).

(2) At the familial level, direct victimization and the perception of maternal inefficacy emerged as relevant predictors, highlighting how the quality of parental practices may act either as a risk factor or a protective element, depending on the degree of consistency and control exercised (Camargo, 2023; Simmons et al., 2020). These findings reinforce the hypothesis of a continuum between experiences of victimization and aggressive behaviors, consistent with theoretical models of the family violence cycle (Gallego et al., 2019). However, in the case of CPV toward the father, the perception of maternal inefficacy was negatively associated with violent behavior, suggesting that different family and relational configurations may modulate conflict dynamics differently toward the two parental figures. One possible interpretation of this finding is that when the mother is perceived as ineffective, the father may assume a more salient or authoritative role within the family system, potentially reducing the likelihood of direct confrontation toward him. Alternatively, this pattern may reflect specific relational dynamics, such as alliance or triangulation processes, in which aggressive behavior is more likely to be directed toward the parent perceived as less authoritative or more emotionally accessible. These interpretations are preliminary and would benefit from further investigation.

(3) At the social level, affiliation with deviant peers predicted CPV toward the mother, whereas perceived family support functioned as a protective factor. Although both variables were significant, their effects were modest, suggesting that during young adulthood family-related processes may play a more prominent role than peer influences in shaping CPV, particularly in contexts characterized by prolonged cohabitation with parents.

An unexpected result concerned the association between female gender and CPV toward the mother, together with the positive correlation with maternal support measures. Although modest, this association may reflect complex relational dynamics in which supportive behaviors coexist with permissive or inconsistent parenting, or where gender-specific communication patterns influence conflict escalation (Cano-Lozano et al., 2024). In line with previous research, indeed, girls tend to report higher levels of reactive aggression and stronger correspondence between experienced and directed violence, particularly toward mothers, suggesting that conflictual interactions may be more emotionally charged and reciprocal within this dyad. Furthermore, greater relational closeness and emotional expressiveness in mother–daughter relationships may, under certain conditions, contribute to the intensification of conflicts rather than their resolution. Given the exploratory nature of these findings, further replication in independent samples is warranted. Moreover, these findings should be interpreted with caution considering the binary operationalization of gender adopted in the present study. By restricting gender to a male/female categorization, it is not possible to capture the complexity and diversity of gender identities, which may play a relevant role in shaping relational and conflict dynamics. It is therefore possible that the observed gender differences reflect, at least in part, a simplified representation of gender-related processes. Future research should adopt more inclusive measures of gender to explore whether and how gender-diverse identities may be associated with different patterns of CPV and family interactions.

### 4.1 Limitations

However, it is important to acknowledge the study's limitations and to clarify their implications for the interpretation of the findings. First, as a cross-sectional study, causal relationships cannot be established; therefore, the observed associations should be interpreted as indicative of relationships rather than directional effects. Moreover, the study relied exclusively on self-report measures, which may be subject to recall and social desirability biases, potentially affecting both the reported prevalence of CPV and the strength of the observed associations.

The use of a convenience sample further limits the generalizability of the findings, as the results may not fully extend to young adults living independently, to clinical or judicial populations, or to cultural contexts characterized by different family structures and emancipation patterns. At the same time, the focus on a non-clinical sample provides ecologically valid insights into a population that remains underrepresented in the literature.

Finally, some measures (e.g., substance use and deviant peer relationships) exhibited relatively low internal consistency, which may have attenuated the observed associations. Unexpected or counterintuitive findings—such as the positive association between maternal support and CPV toward the mother, as well as the role of gender—should therefore be interpreted with caution and require replication in future studies. In this regard, another limitation of the study is the operationalization of gender through a binary categorization (male/female), which does not allow for capturing the diversity of gender identities. This aspect may have influenced the results to the extent that it simplifies a complex variable, especially considering that gender showed weak associations and only in relation to CPV toward the mother. Therefore, it cannot be dismissed that a more accurate measure of gender might have allowed for the identification of additional nuances in its relationship with CPV. Furthermore, this limitation restricts the generalization of the findings to populations with gender diversity. As future lines of research, it is necessary to develop a more inclusive operationalization of gender, as this could reveal more complex relationships between gender and CPV, particularly given that gender identity may interact with relevant risk factors identified in the present study.

Overall, these considerations suggest that the findings should be interpreted within their methodological boundaries, while still providing meaningful insights into the factors associated with CPV in young adulthood.

### 4.2 Future research directions

Despite these limitations, the present study allows outlining several directions for future research. Longitudinal designs would be particularly valuable to determine whether the identified risk factors precede the onset of CPV or emerge because of escalating family conflict. Future studies should also examine potential mediating and moderating mechanisms, such as emotional regulation strategies, attachment patterns, and family communication styles, to better clarify the pathways linking individual vulnerabilities and family dynamics to CPV.

Further investigations could expand the focus on protective factors, including resilience, coping strategies, and external sources of support, to develop a more comprehensive psychosocial profile of young adults involved in CPV. Finally, qualitative research may offer deeper insights into relational processes, particularly in cases where parental support coexists with violent behavior, helping to contextualize quantitative findings and identify clinically relevant dynamics.

### 4.3 Prevention and clinical implications

This study also offers relevant implications for prevention and clinical practice. The findings highlight the complexity of parent–child relationships in young adulthood and suggest the importance of interventions that simultaneously address individual self-regulation difficulties and family dynamics. Prevention programs should include training in emotional regulation and impulse control, for example through cognitive-behavioral techniques and skills-based interventions; alongside psychoeducational initiatives aimed at reducing substance use and increasing awareness of its role in exacerbating family conflict.

At the family level, interventions should focus on promoting consistent and non-impulsive disciplinary strategies while fostering supportive and emotionally attuned parenting practices. Parent training programs and family-based interventions may be particularly effective in improving communication, reducing coercive cycles, and enhancing parental responsiveness. Clinical interventions may also benefit from addressing intergenerational trauma and patterns of victimization, helping families recognize and interrupt cycles of violence.

In addition, given the role of peer deviance and social context, preventive efforts could include community-based programs aimed at promoting prosocial peer relationships and strengthening social support networks. At the same time, strengthening perceived family support and improving communication within the family system may represent key protective factors capable of reducing the risk of CPV during the transition to adulthood.

## Data Availability

The raw data supporting the conclusions of this article will be made available by the authors, without undue reservation.
